# A Rare Facultative Anaerobe Causing Emphysematous Osteomyelitis of the Pubic Bone

**DOI:** 10.7759/cureus.26575

**Published:** 2022-07-05

**Authors:** Samra Iftikhar, Sidrah Iftikhar, Nadeem Ijaz, Mehmood Akhtar, Hina Gul

**Affiliations:** 1 Diagnostic Radiology, Khyber Teaching Hospital Medical Teaching Institution (MTI), Peshawar, PAK; 2 Medicine, Hayatabad Medical Complex Peshawar, Peshawar, PAK; 3 Critical Care, Northwest General Hospital and Research Centre, Peshawar, PAK; 4 Surgery, Khyber Teaching Hospital Medical Teaching Institution (MTI), Peshawar, PAK; 5 Radiology, Khyber Teaching Hospital Medical Teaching Institution (MTI), Peshawar, PAK

**Keywords:** morganella morganii, pubic bone, computed tomography imaging, diabetes mellitus, emphysematous osteomyelitis

## Abstract

Emphysematous osteomyelitis is an uncommon and fatal disease that can only be confirmed with a contrast-enhanced CT scan showing characteristic features of air locules within the bone. It usually occurs in the setting of existing comorbidities and suppressed immune system resulting in widespread bacteremia which may be mono or poly-microbial. Presented here is a case of this disease caused by an unusual anaerobe affecting the pubic bone. This case emphasizes the importance of early imaging as it is crucial for the diagnosis and can facilitate early aggressive management. Moreover, it highlights the importance of early intravenous antibiotics and surgical intervention, which can be life-saving and result in a better outcome in the future.

## Introduction

Emphysematous osteomyelitis (EO) is an exceedingly rare and potentially life-threatening condition, with a reported mortality of above 30%, characterized by intra-osseous gas locules [1]. The exact etiology is unknown, however, several underlying comorbidities, like diabetes mellitus, alcohol abuse, malignancy, sickle cell anemia, steroids and conditions causing immunosuppression can predispose to this disease. Anaerobes or members of the Enterobacteriaceae family are generally the causative agents; however, the infection can have mono or polymicrobial origin [2,3]. The most sensitive and specific imaging modality for the definitive diagnosis of intra-osseous pneumatosis is a computed tomography (CT) scan. Only 38 cases have been reported in the literature so far and no case has been reported in Pakistan to date [4].

## Case presentation

A 72-year-old lady presented with swelling of the left lower limb for the last three days. Initially, swelling of the feet and ankles was noted, however, it progressed upwards involving the left lower limb up to the mid-thigh level. The swelling was associated with pain and low-grade fever. On examination, the left leg was swollen, warm, tender to touch, having increased girth compared to the opposite limb, peripheral pulses were present and neurological examination of the leg was normal. The systemic examination is summarized in Table 1.

**Table 1:** Examination findings on arrival mmHg: millimeter of mercury

Examination	Findings
Blood Pressure (mmHg)	130/80
Heart Rate (beats/minute)	108
Temperature (Fahrenheit)	100
Respiratory Rate (breaths/min)	28
Glasgow Coma Scale (GCS)	14/15
Saturation (SpO2 %)	92
Chest	Bilateral coarse crepitations
Abdomen	Soft, nontender and audible bowel sounds

She was admitted to the medical team and blood investigations and blood culture and sensitivity (C/S) were sent. The patient had type 2 diabetes mellitus and hypertension for the last 10 years for which she had been taking regular medications. Furthermore, she was admitted one month back for the treatment of left arm cellulitis. Significant laboratory investigations are summarized in Table 2.

**Table 2:** Initial lab findings. g/dL: gram/deciliter, mcL: microliter, mg/dL: milligram/deciliter, ng/mL: nanogram/milliliter, mcg/L: micrograms/liter, IU/L: International unit/liter, ELISA: Enzyme-linked immunosorbent assay, HBsAg: Hepatitis B surface antigen, HCV: Hepatitis C virus, HIV: Human immunodeficiency virus, SARS-CoV-2: Severe acute respiratory syndrome coronavirus 2, PCR: Polymerase chain reaction

Investigation	Reference Range	Results
Hemoglobin (g/dl)	11.5-17.5	8.8
White cell count (Í10^3^/mcL)	4-11	10.3
Neutrophils (%)	40-75	54
Lymphocytes (%)	20-40	37
Platelets (Í10^3^/mcL)	150-450	67000
C-Reactive Protein (mg/L)	<5	20.37
Serum Ferritin (mcg/L)	(11-307)	1109
D Dimers (ng/ml)	<500	7344
Bilirubin (mg/dl)	0.1-1	0.37
Alanine aminotransferase (IU/L)	10-50	36
Alkaline Phosphatase (IU/L)	35-104	99
PT (seconds)	12	15
APTT (seconds)	30	32
Urea (mg/dl)	10-50	12.4
Creatinine (mg/dl)	0.4-1.06	0.28
HbA1c (%)	4-6	7
Chest X-ray	Normal	
Electrocardiogram	Normal	
Echocardiogram	Normal	
SARS-CoV-2 PCR	Negative	
Anti-HCV (ELISA)	Negative	
HBsAg (ELISA)	Negative	
Anti-HIV (ELISA)	Negative	

**Figure 1:**
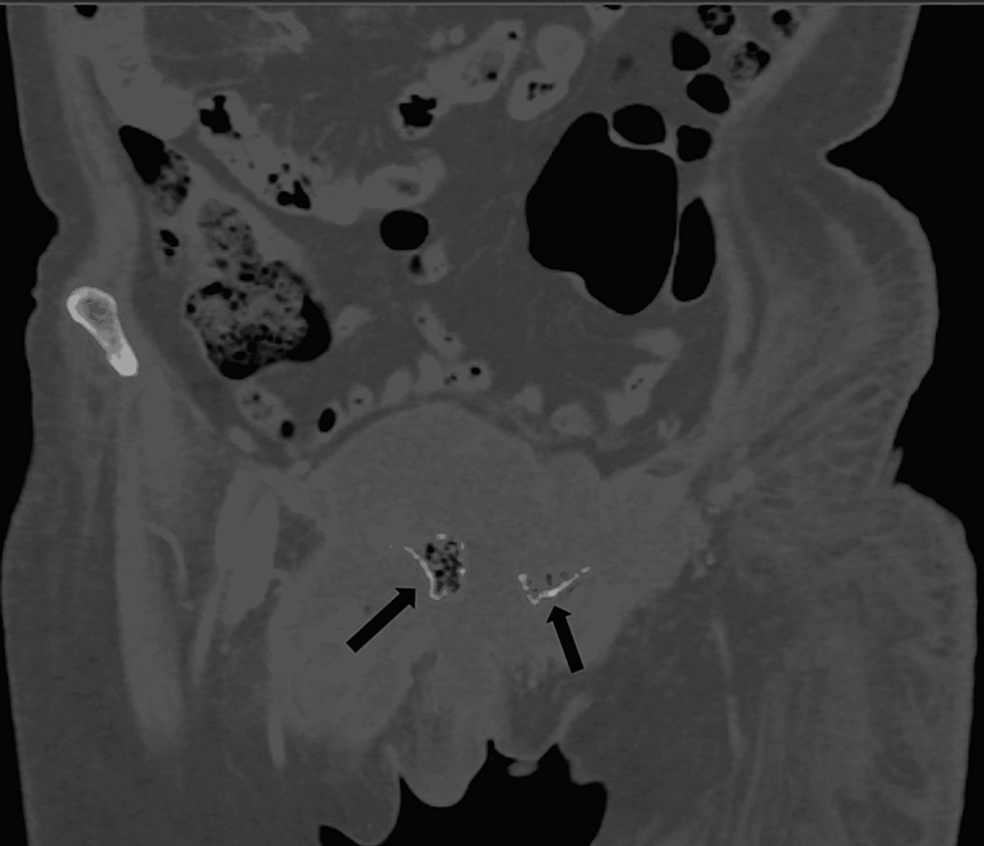
CT abdomen and pelvis (coronal view, bone window) showing clusters of greater than three distinct foci of intramedullary gas with irregularly irregular sizes, giving the classic "Pumice Stone sign" of emphysematous osteomyelitis. CT: Computed tomography

**Figure 2:**
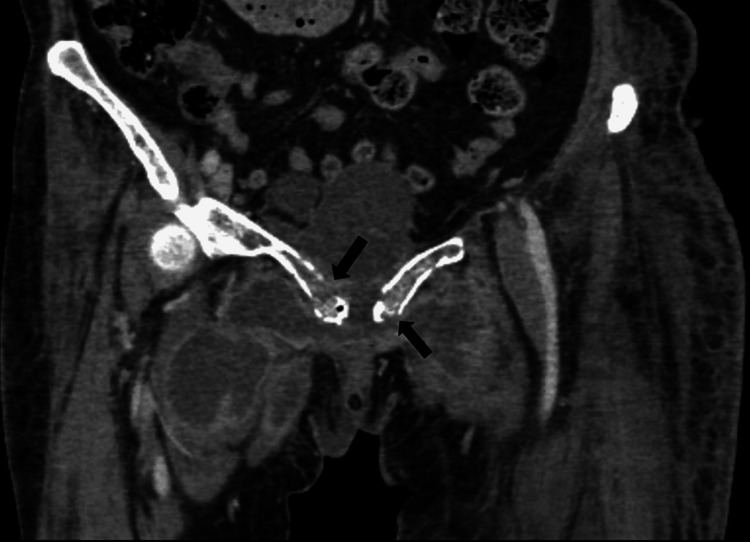
CT abdomen and pelvis with contrast (coronal images, soft tissue window) showing multiple loculated pockets of collection in the perineum, anterior and right hemi-pelvis surrounding the pubic bone. There are cortical erosions of bilateral pubic bones.

**Figure 3:**
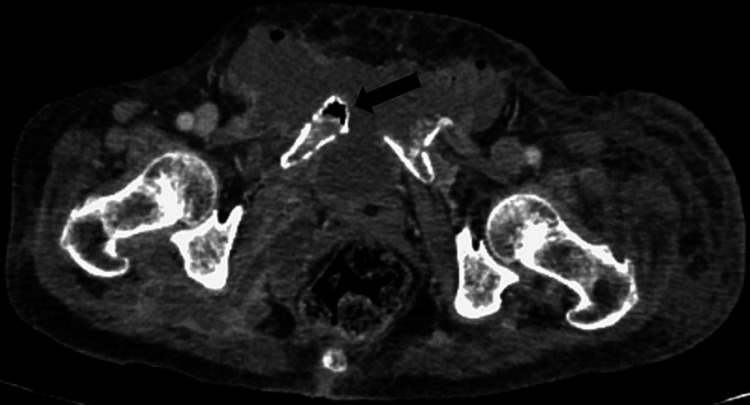
CT abdomen and pelvis (axial section, soft tissue window) showing intra-osseous pneumatosis of the pubic bone.

**Figure 4:**
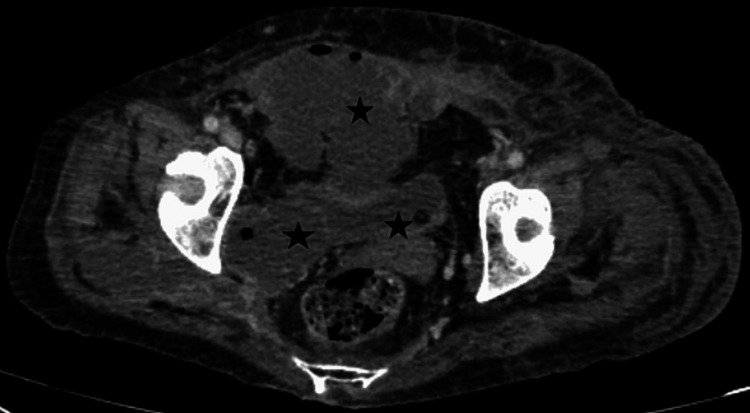
CT abdomen and pelvis (axial images soft tissue window) showing pockets of collections are seen in the right ishio-rectal fossa, extending into the right gluteal region and the superio-medial part of bilateral thighs. Most of these collections have enhancing walls and contain air foci.

**Figure 5:**
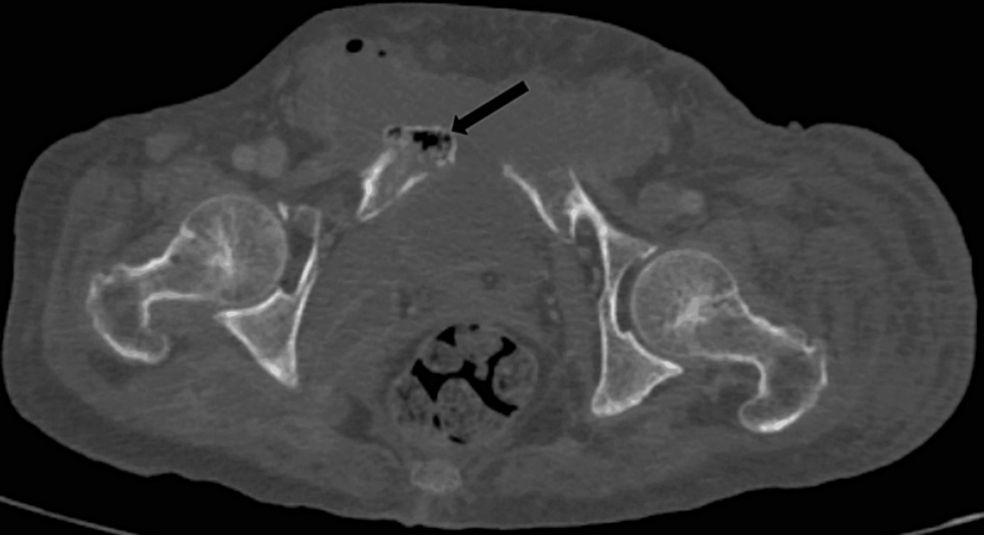
CT abdomen and pelvis (axial section, bone window) showing intra-medullary gas with irregularly irregular sizes, consistent with emphysematous osteomyelitis.

On account of the raised D-dimers and lower limb swelling, deep venous thrombosis was suspected. A Doppler ultrasound was ordered which revealed extensive deep venous thrombosis involving the left iliac vein, femoral vein, and popliteal vein. Further workup for the possible underlying cause of DVT was initiated. CT scan of the chest abdomen and pelvis with contrast was done which revealed multiple irregular patches of collection and connecting septa in the perineum, anterior and left hemipelvis, left ischio-rectal fossa, left perianal region extending to the left gluteal area, and superior-medial part of thighs bilaterally. There was the erosion of adjacent parts of the body and inferior and superior rami of pubic bones and the air were seen in the eroded parts of the bone (Figures 1-5). The air within bone is a pathognomic sign of emphysematous osteomyelitis likely due to extensive infection with spread to adjacent tissue and bone as a result of bacteremia.

**Figure 6:**
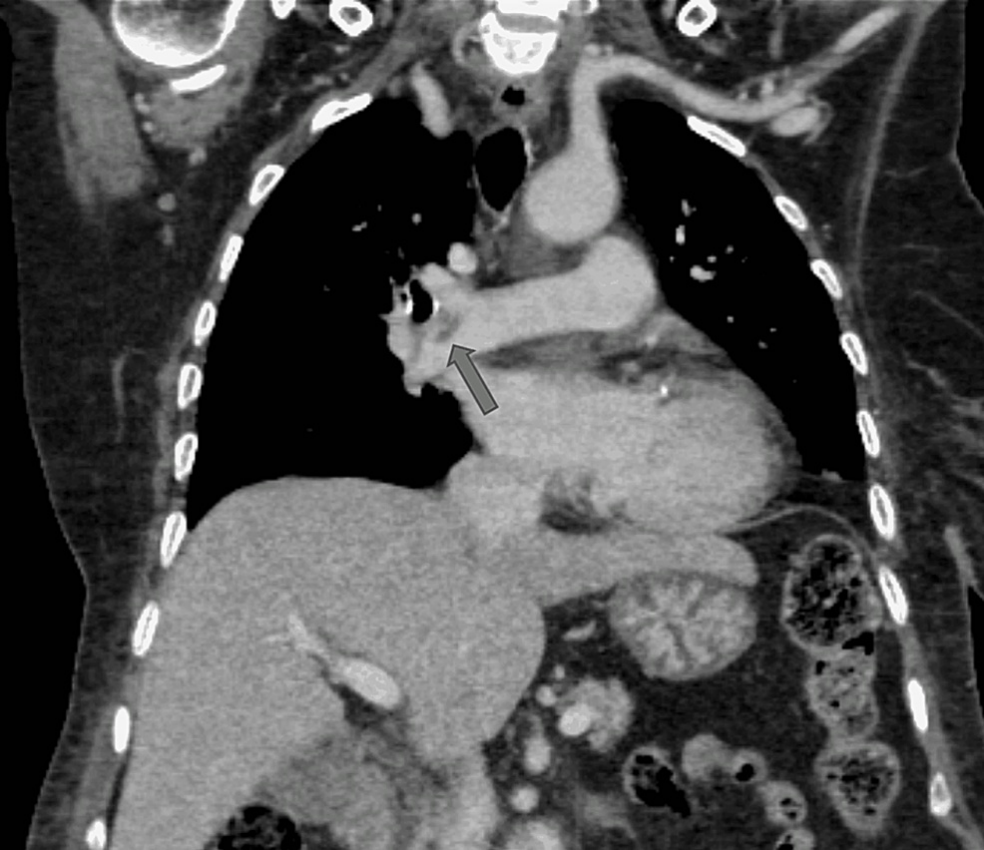
CT thorax (coronal mediastinal window) showing a filling defect in the sub-segmental branch of the right pulmonary artery suggestive of pulmonary embolism.

**Figure 7:**
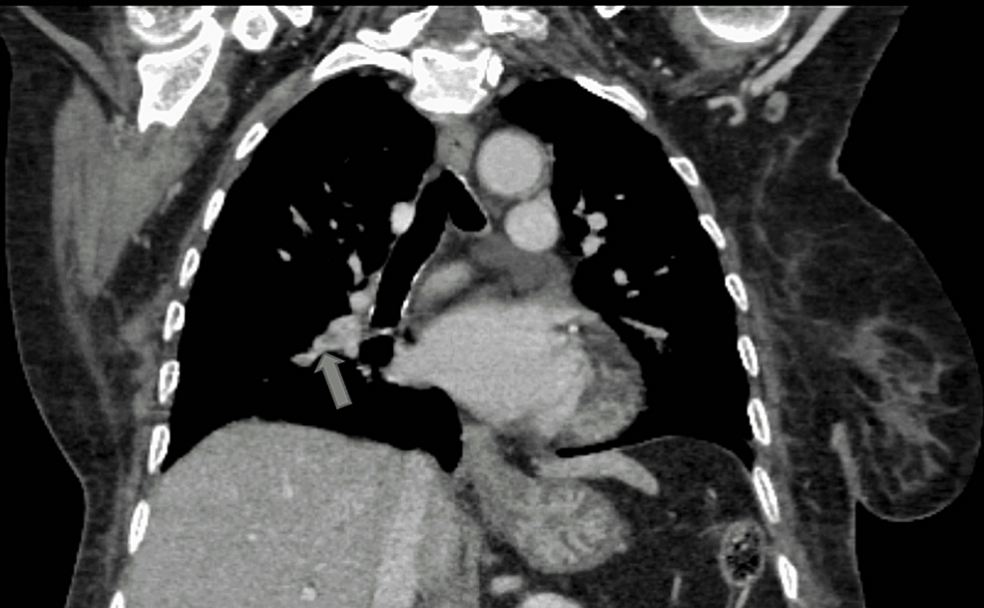
CT thorax (coronal mediastinal window) showing a filling defect at the bifurcation of upper and lower segmental pulmonary branches indicating pulmonary embolism.

CT chest reported a filling defect in the right pulmonary artery extending into lobar branches and multiple small defects within lobar branches of the left pulmonary artery, indicative of pulmonary thromboembolism (Figures 6, 7). Anticoagulation with NOAC and empirical intravenous antibiotics was commenced.

Consultation from the surgery and orthopedic department was undertaken for surgical debridement but due to the critical condition and being a high-risk case, conservative management was preferred. On the 15th day of her admission, the culture report exhibited heavy growth in Marganella morgagni. According to the C/S report, the patient was started on Piperacillin Tazobactam. In the meantime, the patient’s condition deteriorated as she became drowsy and unresponsive. Her GCS was reduced from 14/15 to 9/15. Despite aggressive treatment for three weeks, the patient did not respond well and succumbed to her illness.

## Discussion

Emphysematous osteomyelitis is a rare condition considered a subtype of pyogenic osteomyelitis [5]. It is caused by gas-forming bacteria in most instances and is characterized by locules of air within the bone. These features may be present in the appendicular skeleton as a result of age-related degeneration but when seen in the axial skeleton, they are highly suggestive of emphysematous osteomyelitis. Just like any uncommon infection, certain predisposing factors have to be present for this infection to occur e.g., diabetes mellitus, immunosuppression, trauma, surgery, alcohol abuse, etc. Usually, there is either a contiguous spread of infection or it spreads from a distant focus elsewhere in the body. This can also happen spontaneously without any existing comorbidities requiring urgent surgical intervention and prolonged treatment with intravenous antibiotics. Most patients need to be treated with antibiotics for approximately four to six weeks.

The major obstacle in the management of this disease is having a high index of suspicion as it has many close differentials and it can be impossible to differentiate this condition from other common illnesses in the developing areas of the world. So far, the diagnosis can only be made definitively by performing a contrast-enhanced CT scan revealing air pockets with minimal cortical destruction within the affected bones. “Pumice stone sign” is a characteristic finding and is found in almost all patients having emphysematous osteomyelitis. Thus, confirming the diagnosis relies heavily on radiological findings pointing in the right direction. The significance of this case is two folds. The first one is the rare location of this disease as it has been previously described to occur predominantly in the lumbosacral region or ileum. Still, in this patient, it was found in pubic bone involving the superior and inferior rami. Previously, only one case has reported this disease affecting pubic bone and it had occurred in a setting of infected aortic aneurysm and it involved the femur, pubic bone, and sacroiliac joint [4]. Secondly, the organism isolated as reported by the blood culture report is M. Morgagni, a rare facultative anaerobe predominantly causing hospital-acquired infections. The most common organism implicated in this condition overall has been Escherichia Coli [6-8]. To the best of our knowledge, this facultative anaerobe has not yet been isolated as a cause of this disease. However, some rare organisms have been reported that include Fusobactrum necrophorum, Finegoldia magna, and Actinomyces europeus. The causative agent plays role in disease prognosis as higher mortality has been noted with certain species [9]. In most of these individuals, underlying comorbidities like type 2 diabetes and hypertension are present and they have some preceding infection, surgery, or trauma resulting in sufficient spread of bacteria into the blood culminating in this life-threatening condition. As observed in reported cases, this patient was also having type 2 diabetes mellitus and an episode of left arm cellulitis about a month back.

This patient was having a serious disease in the form of complicated partially treated cellulitis and deep vein thrombosis resulting in pulmonary thromboembolism. This plethora of diseases could explain the critical condition of the patient and the lack of improvement with aggressive intravenous antibiotic treatment and anti-coagulation. Whether the patient could have benefitted from surgical interventions seems unlikely as she was not fit for general anesthesia and those would have to be performed with high-risk consent. She was also having intra-osseous pneumatosis in an unusual site which could have played a crucial role in determining the long-term survival of the patient.

## Conclusions

EO is a radiologically diagnosed entity with very few cases reported in the literature. It is a serious condition and timely diagnosis is central to management. Here, we discussed a patient suffering from this disease having serious co-morbidities and risk factors, where a rare organism caused this infection at a rare site. We suggest that an early thorough evaluation of the patient and having a low threshold for CT scan in such patients is necessary to confirm the diagnosis and initiate immediate therapy to ensure patient survival and reduce mortality.
